# Nondestructive and Fast Vibration Phenotyping of Plants

**DOI:** 10.34133/2019/6379693

**Published:** 2019-06-25

**Authors:** E. de Langre, O. Penalver, P. Hémon, J.-M. Frachisse, M.-B. Bogeat-Triboulot, B. Niez, E. Badel, B. Moulia

**Affiliations:** ^1^LadHyX, CNRS, Ecole Polytechnique, Institut Polytechnique de Paris, 91128 Palaiseau, France; ^2^Institute for Integrative Biology of the Cell (I2BC), CEA, CNRS, Univ. Paris‐Sud, Université Paris‐Saclay, 91198, Gif‐sur‐Yvette cedex, France; ^3^Université de Lorraine, INRA, AgroParisTech, UMR Silva, 54000 Nancy, France; ^4^Université Clermont Auvergne, INRA, PIAF, 63100 Clermont-Ferrand, France

## Abstract

The frequencies of free oscillations of plants, or plant parts, depend on their geometries, stiffnesses, and masses. Besides direct biomechanical interest, free frequencies also provide insights into plant properties that can usually only be measured destructively or with low-throughput techniques (e.g., change in mass, tissue density, or stiffness over development or with stresses). We propose here a new high-throughput method based on the noncontact measurements of the free frequencies of the standing plant. The plant is excited by short air pulses (typically 100 ms). The resulting motion is recorded by a high speed video camera (100 fps) and processed using fast space and time correlation algorithms. In less than a minute the mechanical behavior of the plant is tested over several directions. The performance and versatility of this method has been tested in three contrasted species: tobacco (Nicotiana benthamian), wheat (Triticum aestivum L.), and poplar (Populus sp.), for a total of more than 4000 data points. In tobacco we show that water stress decreased the free frequency by 15%. In wheat we could detect variations of less than 1 g in the mass of spikes. In poplar we could measure frequencies of both the whole stem and leaves. The work provides insight into new potential directions for development of phenotyping.

## 1. Introduction

As emphasized recently in several studies (see [1]) there is an urgent need for new, robust, and reliable phenotyping techniques for plants. Moreover, new traits need to be explored, to give a better knowledge of the phenome in general. So far, most of the explored phenotypes are those that can be accessed by optical means [1, 2]: size, biomass, photosynthetic state, temperature, water content, shape, geometry, and morphometric parameters. Considerable progress has been achieved along these directions, for both indoor and outdoor phenotyping. High-throughput phenotyping (HTP) using optical techniques is now a reality. At the same time, mechanical traits such as the stiffness of a plant, or of part of the plant, are still difficult to obtain. They often require destructive techniques and time-consuming procedures. Still, mechanical phenotypes may be relevant for plant sciences and breeding in two ways. First, mechanical phenotypes can characterize the ability of the plant to resist mechanical loading induced by wind or gravity. But mechanical phenotypes may also give an insight into nonvisible properties or elements of the plant and its constitutive materials (hidden cavity, e.g., pith, change in tissue properties between genotypes or along development, hidden growth, and action of internal diseases) thereby yielding a more complete view of the state of the plant, beyond what can be retrieved through optical means.

Plant biomechanics, where plants are analyzed as mechanical system, has developed in many directions, notably in the field of dynamics; see, for instance, Niklas [3], Speck and Spatz [4], de Langre [5], Gardiner et al. [6], de Langre [7] to cite a few reviews. It has become possible to measure in detail the frequencies characterizing the oscillation of plants, or part of plants, using simple video optical techniques and adequate signal processing [8–10]. Recently, Der Loughian et al. [11] investigated the relation between these frequencies and the size of the plant in* Arabidopsis thaliana* and young* Populus tremula x alba*. They suggested the possibility of using this vibration analysis for phenotyping. Yet, the techniques involved were not adapted to HTP, because of the need of exciting the plant by contact and of the complex signal processing involved; as a consequence their approach was both time-consuming and possibly destructive. Moreover, many of the Digital Image Correlation Techniques (DIC) that have been developed with success for vibration problems (see, for instance, [12, 13]) are not well adapted to the present case of HTP on plants: they aim at both frequencies and modal shapes (a rich information, not needed here) and require high quality optical environment. The use of DIC to analyze the wind induced-motion of a full foliage [14] required lengthy image processing.

Adapting plant vibration measurement to HTP is a challenge as the technique needs to be both nondestructive and fast (say about one minute per plant). Recent works showed promising technical improvements: by exciting leaf motion using acoustic waves and measuring the resulting leaf motion by a laser sensor, Sano et al. [15] showed that water stress did modify the frequency of motion of a leaf. Nondestructive and fast, this technique is nevertheless difficult to apply to whole plants. Shah et al. [16] gave an overview of several other methods available to measure the mechanical properties of stems, but unfortunately all are destructive. In Nakata et al. [17], the first HTP of a plant organ by vibration was demonstrated on* Arabidopsis thaliana* stems. This showed the possibility of HTP by vibrations. But the technique used was still destructive.

In this paper we show how fast nondestructive vibration phenotyping can be done simply. Preliminary tests on several species are presented, exemplifying the possibilities of the method.

## 2. Materials and Methods

### 2.1. Plants

**Figure 1:**
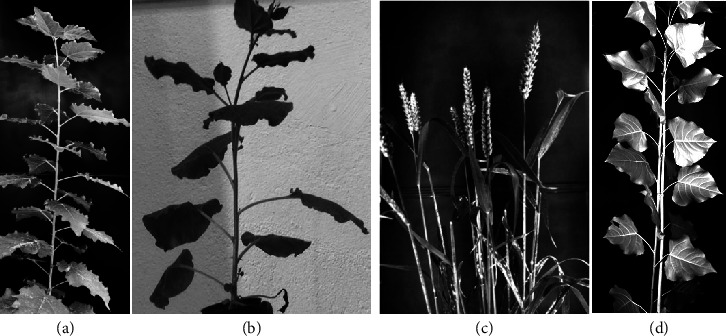
Typical plants used in the experiments: (a) poplar, (b) tobacco, (c) wheat, and (d) tutored poplar.

Poplar: the poplar young plant used in the present work is part of the set described in Niez et al. [18]. The conditions of their culture are briefly recalled here. Hybrid poplars (*P. tremula x P. alba*, clone INRA 717-1B4) were clonally multiplied* in vitro* on 1/2 strength Murashige and Skoog medium. After two months of potting and acclimation, young trees were selected according to their homogeneity and transferred to a greenhouse at 22±1°C (day) and 19±1°C (night) under natural light (Clermont-Ferrand, France, N 45.77° E 3.14°). They were planted in 10L pots, filled with a substrate composed of one-third black peat and two-thirds local clay-humic Limagne soil. Then, the tree used in the present experiment was grown for 7 weeks in well-watered condition and received 200 g of nutrient solution per week. It is shown in Figure 1(a). The height of the tree was about 65 cm and its diameter was about 7 mm at 20 cm above the stem base (the cross-section of the stem was totally symmetrical at the time of the vibration measurements). At the time of the test the age of the tree was 15 weeks after cloning.

Tobacco:* Nicotiana benthamiana* seeds were sown on pit pellets (Jiffy-7), with one seed per pot. They were cultivated in long day conditions (8-hour darkness, 16 h00 light) at 24°C during 22 days and then at 21°C. Two groups of 40 plants were randomly selected on day 41. Vibration phenotyping tests were done from days 42 to day 45 after sowing, corresponding to a stage before flowering. The control group was daily watered and tested a few hours after. The water-stressed group was not irrigated on days 41, 42, and 43 but reirrigated on day 44. The first test on the water-stressed group, day 42, was done 20 hours after the last watering. The last test on the same group, day 45, was done 20 hours after the rehydration of day 44. At the time of tests the plants were about 20 cm high, with 12 to 14 developed leaves. A sample plant is shown Figure 1(b).

Wheat: seeds of winter wheat (*Triticum aestivum* L., cv Recital) were sown in 50 mL plastic pots (two seeds per pot) placed in greenhouse. The growing conditions are detailed in Baillot et al. [19] and summarized here. When the third leaf emerged, the plants were transferred into a vernalization chamber maintained at 5°C with a 8 h photoperiod. After 8 weeks, the plants were transplanted into 4L pots (2 plants per pots), filled with a compost enriched with fertiliser N-P-K (9-12-16) and kept in a greenhouse (located in Clermont-Ferrand, France, N 45.77° E 3.14°). The air temperature was 19°C/15°C (light/dark) with 16 h/8h (light/dark) photoperiod. The plants were watered daily with nutrient solution. The number of reproductive tillers (with spikes) ranged between 8 and 16 per pot. Most flowering of the spikes occurred around 130 days after sowing. Two pots (four plants in total) were transferred into the lab for the present experiments, respectively, 155 and 160 days after sowing. At that time, the majority of grains in the spikes were in the filling phase. These control plants were then modified by the addition of masses of plasticine of 0.5g on each ear and then of 1.0 g and tested on the same day. A sample is shown Figure 1(c).

Tutored Poplars:* Populus canadensis* cultivar Carpaccio woody cuttings were obtained from clonal propagation and were planted in 10 L plastic pots filled with a 1:1 (v/v) mixture of peat and sand, amended with a slow release fertiliser (4 g l^−1^ of Nutricote T100, 13:13:13 NPK and micronutrients; FERTIL S.A.S, Boulogne Billancourt, France) and 1 g l^−1^ CaMg(CO_3_)_2_. Plants were grown in a glasshouse located at Champenoux, France (48°45′09.3′′N, 6°20′27.6′′E), under natural light conditions. Environmental conditions in the greenhouse were affected by weather conditions, but the temperature was maintained between 15 and 28°C. The plants were tutored during growth and the present test, with a bamboo stick where the stem is attached every 20 cm. At the day of the experiment the plants were three months old after cloning. Poplar was 1 m80 high and had 35-40 leaves. Leaves that were phenotyped were in the middle of the stem and were all mature. A sample is shown Figure 1(d).

### 2.2. Vibration Phenotyping System

**Figure 2:**
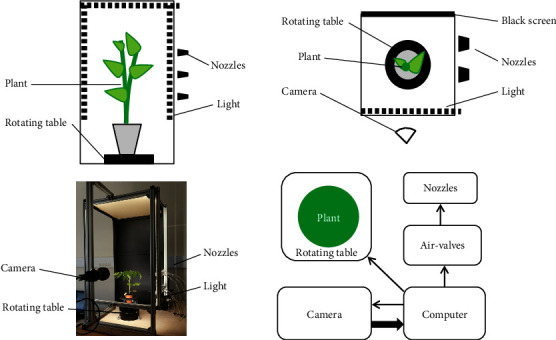
Phenotyping system. Left: side view. Right: top view and general organization

A general scheme of the system is shown in Figure 2, and a corresponding patent is described in de Langre et al. [20]. The plant is excited by a short air pulse (50 to 500 ms). This pulse is generated using electrovalves SMC VX210HG, connected to compressed air stored in a tank under a pressure of 7 bars. The opening of the valves during a short time lapse is controlled by a central computer. The electrovalves, when open, send air to a series of flat nozzles placed laterally to the plant. For a given series of experiments, the duration of the air pulse is tuned in such a way that the air-dragged motion of the plant can be detected (of a cm or so) while remaining within the elastic range. The plant is lit by LEDs (180 lumen/m) placed on the frame around the plant. A Luminera Lt225 camera with 8 mm focal length is placed at a distance such that the whole plant is in the field of view. The free motion of the plant after the air pulse is filmed at 100 fps and 2-Mpixel image definition, during 3 to 8 seconds. The images are transformed from RGB to grayscale and recorded. A shot is defined as the sequence of an air pulse followed by the free oscillation of the plant. The plant is placed on a rotating table that allows a 30° rotation between shots or series of shots. Depending on the species and the growth stage, the system may be adapted in terms of size, number of nozzles, durations of air pulses, and of video recording.

### 2.3. Signal Processing

**Figure 3:**
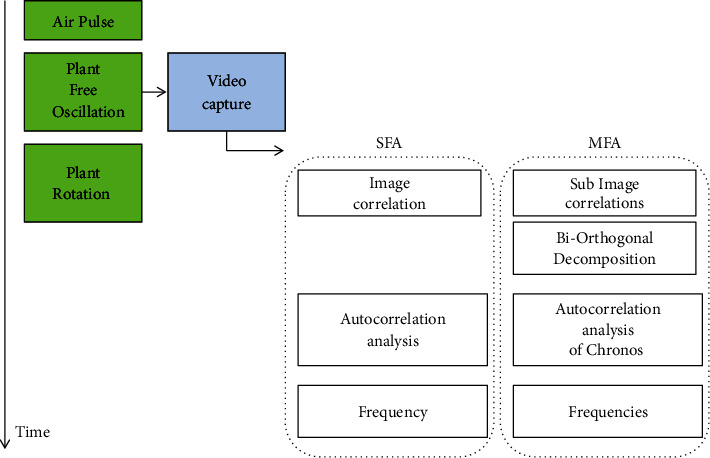
Time sequence of the phenotyping process.

The aim of the signal processing developed for this device is to derive a robust estimate of the frequency, or frequencies, of the plant oscillation from the video capture of the motion, within a few seconds. Two distinct procedures may be used; see Figure 3. The single frequency analysis (SFA) is well adapted for plants where a single dominant mode of vibration exists in the motion, while the multiple frequency analysis (MFA) is better adapted for plants where several frequencies of close values are present.

**Figure 4:**
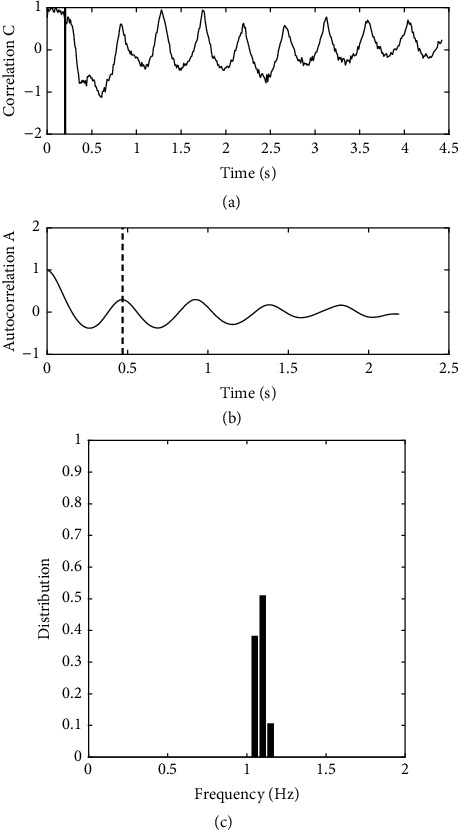
Vibration tests on a single poplar. Typical changes in time of the variables obtained in the single frequency analysis (SFA). (a) Image correlation coefficient C(t). The vertical bar shows the end of the air pulse. (b) Autocorrelation function of this correlation, A(t). The vertical dashed line indicates the value of T the first maximum of A(t). (c) Distribution of frequencies derived in 48 tests, over 12 angles.

In SFA, for each image *I*(*t*_*i*_), a correlation coefficient is computed as

(1) $$\begin{array}{c} C\left(t_{i}\right)=\frac{I\left(t_{i}\right)*I_{0}}{I_{0}*I_{0}}, \end{array}$$

where *I*(*t*_*i*_) is the current image of the plant at time *t*_*i*_ after the air pulse, *I*_0_ is an image of the plant just before the air pulse, and *∗* denotes the sum of the pixel per pixel product of images. Over time *C*(*t*_*i*_) varies periodically as the plant oscillates (see Figure 4(a). The autocorrelation function of *C*, *A*(*t*) is computed starting from the end of the air pulse. The frequency of the motion of the plant is finally derived as

(2) $$\begin{array}{c} f=\frac{1}{2T}, \end{array}$$

where T is the time of the first maximum of *A*(*t*), corresponding to half a period of oscillation; see Figure 4(b). A test for consistency is applied on *f*: if it is close to 1/*T*_*film*_ where *T*_*film*_ is the length of video capture, the measure is considered as failed, as the maximum of the autocorrelation function does not give information on periodicity in that case. Failures to measure appear in less than 5% of the tests. They are generally related to bad interaction between the air-jet and the plant.

In MFA, a more advanced processing is used, based on Bi-Orthogonal Decomposition (BOD). This general method of field decomposition has already been applied to analyze the motion of plants obtained by video capture in several cases such as crops canopies [9], trees [10],* Arabidopsis thaliana* [11], and foliage [14]. In these papers, the video capture was first treated by a Digital Image Correlation technique to derive the velocity field of the plant and its evolution in time. Assuming that this evolution was the result of a superposition of the evolutions of orthogonal vibration modes in response to the excitation, the BOD was used to identify in the space-time signal the dominant features in space (the mode shapes) and in time (the response of the mode). The velocity field *V*(*M*, *t*) was thus decomposed as

(3) $$\begin{array}{c} V\left(M,t\right)=\sum_{p}J_{P}\left(M\right)K_{p}\left(t\right), \end{array}$$

where *J*_*p*_(*M*) are the mode shapes (or topos) and *K*_*p*_(*t*) are the response of the mode (or chronos). In those cases the BOD was closely related to the modal decomposition. Here, we seek to extract from the video capture the main frequencies that compose the signal, if several contribute. Using the same method as in the papers cited above would be prohibitive in terms of computational time and in fact useless we do not need the mode shapes. The evolution of the simple correlation coefficient *C*(*t*_*i*_) defined above does show several frequencies, as illustrated in Figure 6(a), but the information contained in this simple signal is too poor to accurately measure them. To retrieve more information from the video capture without computing the velocity field all over the plant, the images *I*(*t*) are here divided in N subspaces (typically 16*∗*16). In each subspace a correlation coefficient *C*_*k*_(*t*) is derived from the set of images in that subspace, *I*_*k*_(*t*) using (1). To extract the dominant features the vector [*C*(*t*)] formed with these N correlation coefficients is then analysed using the Bi-Orthogonal Decomposition. This leads, by decomposition, to a series of orthogonal modes, each mode being characterized by a shape vector (or topos) [*J*_*p*_] and a time evolution (or chronos) *K*_*p*_(*t*) such that

(4) $$\begin{array}{c} \left[C\left(t\right)\right]=\sum_{p}K_{p}\left(t\right)\left[J_{p}\right]. \end{array}$$

A topos [*J*_*p*_] is the equivalent in terms of image correlation, of a motion according to one vibration mode. It is defined in the whole space, by a scalar value in each subspace. The corresponding chronos *K*_*p*_(*t*) is the time evolution of the motion along this mode. The modes can be ranked in terms of decreasing contribution to the motion, and only the 5 dominant modes for each shot were considered in the present paper, as it was found that other modes were then not accurately measured. To derive the corresponding 5 frequencies, the same autocorrelation procedure as in SFA can then be applied on the *K*_*p*_(*t*), in place of the *C*(*t*). An example of a Chronos *K*_*p*_(*t*) is shown in Figure 6(b), with a clear frequency of oscillation. Tests on the dependence of these frequencies on the number of subspaces N showed that frequencies lower than 0.6 times the mean frequency are N-dependent and are actually half-frequencies caused by aliasing. They are consequently removed before outputting the results. All other frequencies did not depend much on N, provided N was larger than 4*∗*4.

### 2.4. Statistical Analysis

Histograms, means, and standard deviations were obtained by the corresponding Matlab functions. Normalized Anova one-parameter tests between the two groups of the Tobacco study were performed using the anova1 Matlab function. In the text and the figures, “distribution” refers to “probability distribution function.”

### 2.5. Mechanical Model

To model the effect of mass on frequencies, the elementary mass-spring model of dynamics is used [21]: a frequency *f* varies as $1/\sqrt{M}$ where *M* is the mass of the system. As a result, if an additional mass *m* is added, the frequency varies as

(5) $$\begin{array}{c} \frac{f\left(m\right)}{f\left(0\right)}=\sqrt{\frac{M}{M+m}}. \end{array}$$

### 2.6. Tests

 See Table 1.

## 3. Results

### 3.1. Poplar

One poplar is tested over 12 angles of rotation of the table (0 to 330 degrees), using SFA. The typical changes of C(t) and A(t) with time are shown in Figures 4(a) and (b), respectively. The correlation C shows a clear oscillation with time. It decreases after the air pulse, as the plants bend away from its initial position, and reincreases as the plants spring back. It comes back to almost its initial value when the plant passes through its initial vertical position. Note that the first oscillation contains more complex evolutions, corresponding to other modes of motion (torsion of the whole plant, motion of leaves, etc.). The later oscillations are regular in period and decreasing in amplitudes. The autocorrelation function A(t) of this whole time-series shows a well-defined damped oscillator behavior. This allows defining the period, here T=0.47s, and the frequency f= 1.06 Hz, derived from this shot. The frequencies corresponding to 48 shots (4 shots per angle, as the plant is rotated) are given in Figure 4(c). A mean frequency of 1.09 Hz is derived with a standard deviation of 0.03 Hz. It corresponds to a global bending of the plant, as in Der Loughian et al. [11]. Note that, following vibration theory, two distinct frequencies should be seen if the system is not perfectly circularly symmetric, corresponding to orthogonal directions of motion. Here, the plant is mechanically symmetric enough that only small variations of frequency can be found when the angle of excitation is changed. The distribution of frequencies shows both a good reproducibility of the tests and a well defined mean frequency for this plant. In that test, about 10 seconds is needed per shot. Consequently, a reasonable approximation of the distribution of frequencies would be obtained in 2 minutes, with one shot per angle only.

### 3.2. Tobacco: Effect of Water Stress

**Figure 5:**
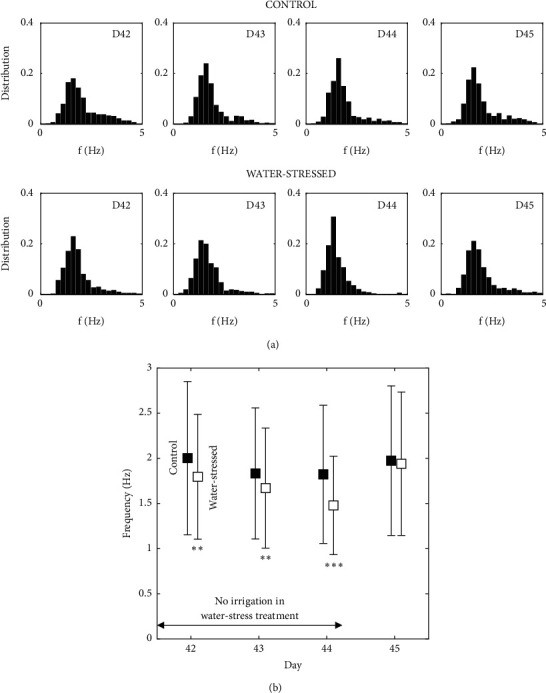
Impact of water stress on the frequencies of oscillation of* Nicotiana benthamiana*. (a) Distribution of the frequencies from days 42 to 45. Control and water-stressed groups. (b) Evolution of the means and standard deviation of theses frequencies, showing the effect of the water stress, and a recovery after rehydration, day 45.

The results of measured frequencies in control and water-stressed groups of Tobacco plants are compared over four days. Each day, in each group, 480 frequencies are obtained through 12 shots on each of the 40 plants. The resulting distributions of the frequencies are shown in Figure 5(a). The distribution of frequencies shows a pronounced peak in each case. On the two groups, the mean and standard deviation of these frequencies are shown, Figure 5(b) over the four days. Frequencies are statistically highly different between the two groups during the water stress days, 42 (20 hours of water stress) to 44, (p < 0.01, labeled *∗∗*, p < 0.001, *∗∗∗*). This is consistent with the observation of some leaf wilting in the stressed group which suggests a decrease in turgor pressure and therefore in the plant stiffness. On Day 45, 20 hours after reirrigation the measured mean frequency of the water-stressed plants recovers that of the control plants (p > 0.5). These tests show the possibility of relevant statistical studies. A frequency is obtained every 10 seconds. The 960 data points for each day are obtained in about 3 hours.

### 3.3. Wheat: Multistems Dynamics and Effect of Ear Mass

**Figure 6:**
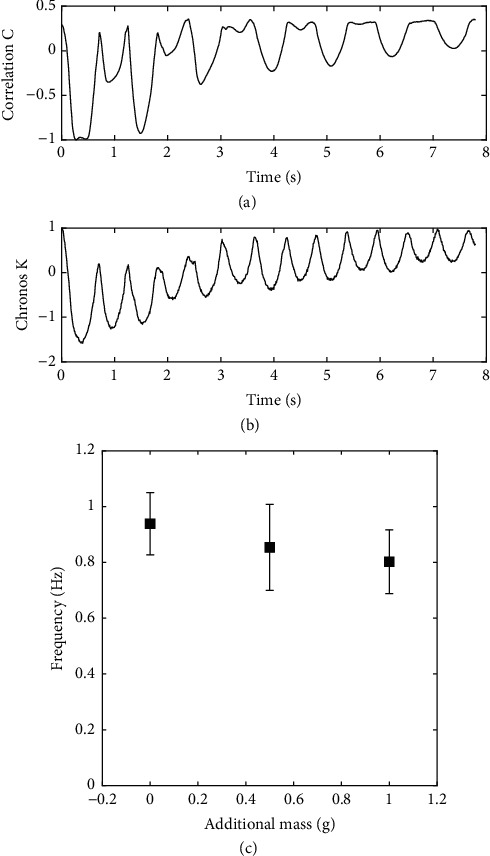
Vibration tests on wheat. (a) Typical time-course of the correlation coefficient C(t), showing a multiple frequency content. (b) Typical time-course of a chronos Kp(t) of one of the dominant modes, obtained by BOD. (c) Effect on the frequency of the additional masses on the ears. The bars correspond to the standard deviation.

For more complex plants with multiple stems, such as wheat with about 20 stems (tillers) per pot, the simple correlation coefficient C(t), as defined in SFA, shows a multifrequency evolution, as illustrated in Figure 6(a). Consequently the MFA method must be used. The simple time-course of a chronos along the same test, Figure 6(b), can then be used for computing a frequency, using the autocorrelation coefficients of this chronos. As described in Materials and Methods, five frequencies were then obtained for each shot, every 30 seconds. About 120 frequencies were computed for each group; see Table 1. The corresponding means and standard deviations are shown in Figure 6(c), as a function of the mass added on each ear. The changes in the frequencies due to the addition of masses are statistically relevant (p < 0.001 between the three groups). The additional masses of 1g induce a decrease of the frequency of about 15%. Using the model for the dependency of frequency with mass, the ear mass can be inferred to be about 2.7g, which is consistent with published data at that growth stage [22].

### 3.4. Tutored Poplars: Leaves Dynamics

**Figure 7:**
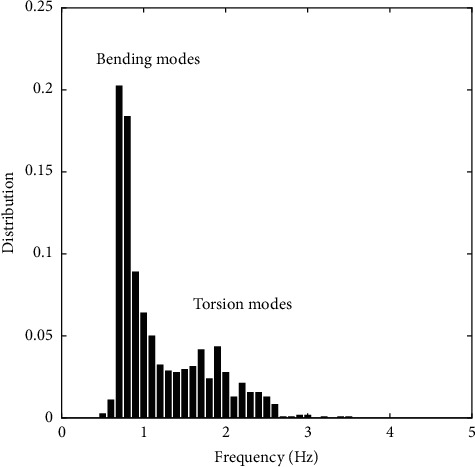
Vibration tests on leaves of tutored poplars. The dominant peak corresponds to bending of the petioles. The secondary peak, near 1.8 Hz, probably corresponds to torsion of the petioles; see Niklas [3].

Because of the tutoring, Figure 1(d), the bending mode of the stem that was observed previously is prevented, and only the foliage moves. A large number of leaves (about 20) are set into motion at each shot. As 5 frequencies are obtained from the MFA at each shot, a good approximation of the distribution of the frequencies present in the whole foliage requires a larger number of shots. This is done here with 120 shots per plant, with a shot every 15 seconds.  Figure 7 shows a distribution with two peaks, a dominant, and a secondary one. By a closer observation of videos, the dominant one, near 0.7 Hz was found to be related to motions involving mainly bending of the petioles. The less pronounced secondary peak, near 1.8 Hz, seemed to correspond more to torsion of the leaves around the petiole axis. These two motions have been observed by Niklas [3], using standard low-throughput methods on poplar leaves with petioles of a few centimeters. Note that in the present test the bending modes clearly dominate the motion, in the sense that their frequencies appear much more frequently in the five most energetic ones derived from MFA. This is due to both the type of excitation, uniform on the foliage, and the rather small amplitude of deformation torsion causes in the images.

## 4. Discussion

We have developed and tested a new approach for vibration phenotyping of plants. In the rapidly developing field of phenotyping methods the present one is innovative and promising in several aspects.

In comparison with existing methods in phenotyping by vibration, such as the most advanced one by Nakata et al. [17], it has some specific advantages. First it is nondestructive: this is a major feature that allows following the characteristic of a group over time, as we have shown here on the tests on Tobacco. Second, this method is applicable to a large variety of plant species, as we showed here with plants of very contrasted morphologies such as wheat and poplars. Further tests, not reported here, successfully involved* Arabidopsis* inflorescence stems and Tomato shoots. For all the plants morphologies, the same system has been used, with adjustment of the air-blowing parameters, and if needed, of dimensions. This versatility is related to the simplicity of the system that uses compressed air, electrovalves, and standard cameras. Third, this method is able to take into account complex dynamics, involving several stems, as shown in wheat, or several leaves, as shown in the tests on tutored poplars. Finally, the method is fast: relevant information on a plant can be obtained by several shots from several angles, in one or two minutes. This includes the signal processing to derive the data (frequencies), so that no batch processing is needed. No specific data storage is involved, and the system can run continuously: it was run without stopping during 4 days on the same plant (data not shown). In practice, using the method for larger groups of plants (hundreds) can be done by simply inserting the device in a HTP platform, where the plants are loaded and unloaded automatically, and by transferring the measured frequencies to a platform data storage. The parameters for the shots (pulse duration, film duration) can be identical for all plants and need only to be changed when the plants have significantly changed. Therefore this system appears suitable for high-throughput phenotyping.

It is expected that the method can be further improved in many of its characteristics. The signal processing, as it is, can be further optimized in speed and accuracy. In terms of speed, several tasks such as the computation of the correlations can actually be done in real time during the free oscillation of the plant. Only the Bi-orthogonal Decomposition and the computation of the autocorrelations require that the video capture is ended; but these are lighter tasks. Using such real-time computation, the duration of one shot can probably be halved. Of course, the time of free oscillation, which is of several seconds if the frequencies of interest are of the orders of one Hz, is incompressible. In terms of accuracy, improvements can be brought by simple optimizations of the video capture length, or by the use of other methods of signal processing, such as Dynamic Mode Decomposition. The simplicity of the system also allows improvements in terms of number of cameras and angles of view, or of nozzles. Moreover, from the same tests, other characteristics could be computed simply such as damping.

More generally, the relevance of frequencies of oscillation as phenotypical traits can be discussed. From a biomechanical point of view, frequencies are known to depend on geometries (dimensions and topology), on the mass of tissues and its distribution in the plant, and on the stiffness and its distribution. Geometries are well accessible now by optical means, but it is difficult to access to masses and stiffness in a nondestructive and fast measure.

In the present work the measured changes in frequencies were the consequence of changes in one single trait (stiffness in the tests on tobacco, or mass in the tests on wheat). The practical question of how to retrieve a specific trait (mass, size, stiffness) from frequencies when several vary simultaneously, is an important one, still to be explored. The answer would probably rely on biomechanical models of the dynamics of the plants or parts of plants. For instance, as discussed in Der Loughian et al. [11] for whole plants (*Arabidopsis thaliana* and poplar), or in Niklas [3] for individual leaves (poplar), the dependence of the frequency *f* on the size *L* can be established for a given group and compared to simple biomechanical models of beams in bending for the stem or for the petiole, respectively. In these two cases a quantity like *f∗L* was approximately a constant over the samples. Any change in this quantity would have to be associated with a change in either local bending stiffness or masses [7]. For the whole plant, the mass to be considered would be the mass per unit length, *M*/*L*, and for a single leaf it would be the mass of the lamina, which varies as the lamina area. As the local bending stiffness integrates both geometrical effects (stem or petiole diameter) and tissue elastic properties, the two cannot be distinguished unless some local geometrical traits are measured. To summarize, measuring frequencies does bring additional information that is not redundant with other accessible information but does not give direct information on one single trait: by combining with other traits it may still be useful to estimate traits difficult to measure.

The method is also capable of providing a nondestructive and easy measurement of the time-course of the effect of water stress of the inner state of plant tissues, suggesting that this method could be a valuable complement to the estimates of traits related to water-stress tolerance [23]. The method needs now to be used more systematically to demonstrate actually phenotyping on the scale of larger groups of plants and under more controlled stress conditions. This is under current investigation.

## Figures and Tables

**Table 1:** Summary of the tests.

Plant	Groups	Plants (pots) per group	Days	Angles	Shots per angle	Frequency Analysis	Film duration (s)	Air pulse duration (ms)
Poplar	Control	1	1	12	4	Single	4.5	200

Tobacco	Control	40	4	12	1	Single	3	60

	Water Stressed	40	4	12	1	Single	3	60

Wheat	Control	2	1	12	5	Multiple	8	400

	Mass 0.5g	2	1	12	5	Multiple	8	400

	Mass 1g	2	1	12	5	Multiple	8	400

Tutored poplar	Control	2	1	2	60	Multiple	3	100
